# Further variation of the adulticide protocol for the treatment of canine heartworm infection: can it be even shorter and cost less?

**DOI:** 10.1186/s13071-022-05625-9

**Published:** 2023-04-28

**Authors:** Karen Ann Ward, Linda Susan Jacobson, Aveline Baldasan Lacaden, Kelly Ann Harrison

**Affiliations:** 1 Toronto Humane Society, 11 River Street, Toronto, ON M5A 4C2 Canada; 2 University of Florida College of Veterinary Medicine, 2015 SW 16th Ave, Gainesville, FL 32608 USA

**Keywords:** Heartworm, *Dirofilaria immitis*, Shelter, Adulticide, Melarsomine, Doxycycline, Modified protocol

## Abstract

**Background:**

This retrospective study evaluated modified three-dose melarsomine treatment protocols in a shelter setting and compared them to the American Heartworm Society (AHS)-recommended protocol.

**Methods:**

As compared with the AHS protocol, the shelter protocols utilized doxycycline 10 mg/kg once daily (SID) or twice daily (BID), and varied the time from initiation of doxycycline (day 1) to the first melarsomine injection (M1). Dogs were retrospectively grouped based on the shelter’s current protocol (M1 on day 14; Group A) and the AHS protocol (M1 on day 60; Group C), allowing a week on either side of the target M1 day. Treatments that fell outside these ranges formed two additional treatment groups (Groups B and D). Respiratory complications were defined as respiratory signs requiring additional treatment, and were statistically compared for Groups A and C. New respiratory signs and gastrointestinal (GI) signs were compared between dogs receiving SID or BID doxycycline.

**Results:**

One hundred fifty-seven dogs with asymptomatic or mild heartworm disease at presentation were included. All dogs survived to discharge. There was no statistically significant difference between Groups A (*n* = 79) and C (*n* = 27) for new respiratory signs post-melarsomine (*P* = 0.73). The time to M1 for 14 dogs that developed new respiratory signs was a median of 19 days, compared with 22 days for 143 dogs without new respiratory signs (*P* = 0.2). Respiratory complications post-melarsomine were uncommon. New respiratory signs post-melarsomine occurred in 10/109 (9.2%) dogs receiving SID doxycycline and 4/48 (8.3%) dogs receiving BID doxycycline (*P* > 0.999). GI signs prior to M1 were recorded for 40/109 (36.7%) dogs receiving SID doxycycline and 25/48 (52.1%) receiving BID doxycycline (*P* = 0.08). Forty-four follow-up antigen test results were available; all tests performed > 3 months after the third melarsomine injection were negative.

**Conclusions:**

This study provided support for initiating melarsomine after 14 days of doxycycline and for a lower doxycycline dose. Shorter and less expensive treatment protocols can increase lifesaving capacity and improve quality of life for shelter dogs by reducing the duration of exercise restriction and length of stay.

**Graphical Abstract:**

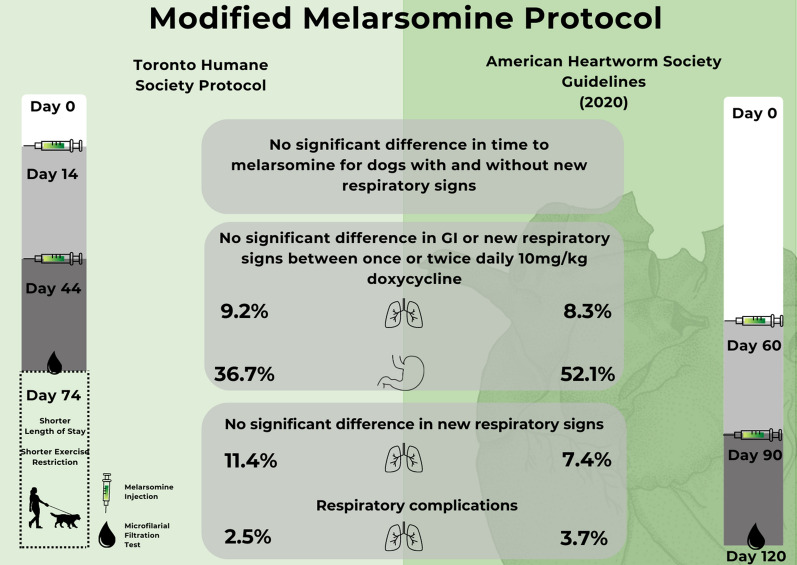

## Background

Melarsomine is the only approved adulticide treatment for *Dirofilaria immitis,* the filarial nematode that causes heartworm infection (HWI). Current treatment recommendations aim to eliminate immature and adult worms while minimizing adverse events following worm death. A three-dose series of melarsomine, together with doxycycline and a macrocyclic lactone (ML), is currently the safest and most effective treatment [1–4]. The American Heartworm Society (AHS) guidelines recommend administering a ML at the time of diagnosis, together with doxycycline 10 mg/kg administered orally (PO) twice daily (BID) from days 1 to 28 [1]. This is followed by the first melarsomine injection (M1) at day 61 post-diagnosis, and the second and third injections (M2/3) a month later, spaced 24 h apart [1]. A course of prednisone is recommended at the time of the melarsomine injections, to reduce pulmonary inflammation associated with worm death.

Doxycycline is used to combat the endosymbiont *Wolbachia* bacteria that coexist with all life stages of *D. immitis*. Pulmonary inflammation following melarsomine is markedly ameliorated by prior treatment with doxycycline and an ML [5–7]. Loss of *Wolbachia* also weakens the worms and decreases their ability to reproduce [8–11]. The currently recommended 30-day interval between completion of the course of doxycycline and M1 is hypothesized to allow time for the *Wolbachia* surface protein and other metabolites to be cleared [1]. The delay may also further weaken the worms and render them more susceptible to melarsomine [1]. Delaying melarsomine was previously recommended to avoid a “susceptibility gap,” during which some heartworms might be too young to be susceptible to melarsomine, but too old to be susceptible to MLs. Adjunctive treatment with an ML has been argued to close this “gap,” and a delay in initiating melarsomine is no longer considered necessary on these grounds [12].

Bowman and Drake [12] have suggested initiating melarsomine treatment as close as possible to the time of diagnosis. The rationale is that the 60-day delay allows an exponential increase in worm volumes, with the potential for greater pulmonary pathology and more severe pulmonary thromboembolism (PTE) post-melarsomine. A delay could also allow some worms to become more refractory to treatment as they age [12]. However, it is important to administer doxycycline prior to melarsomine, to reduce the pulmonary inflammation and PTE that result from worm death [6, 13]. Little research has been published to specifically support the 60-day delay or alternative timing, with only one study directly addressing this question [13]. That study supported initiating melarsomine at day 30 instead of day 61 post-diagnosis.

There is a lack of consensus regarding the optimal doxycycline dosage regimen for HWI, with support for 10 mg/kg once daily (SID), 5 mg/kg BID, or 10 mg/kg BID [1–3, 14, 15]. Doxycycline or minocycline dosed at 5 mg/kg BID was associated with fewer gastrointestinal (GI) side effects than 10 mg/kg BID [14]. GI side effects may reduce drug absorption and efficacy, as well as reducing owner compliance. Cost considerations are also important. Few studies have evaluated respiratory signs in dogs treated with melarsomine and doxycycline, or compared SID and BID doxycycline in dogs with HWI.

Canine HWI represents a challenge for animal welfare organizations (AWOs) [16, 17]. Treatment requires a significant investment of resources that can exceed capacity for care and may result in compromised welfare [18]. Treatment delays and high costs have a direct negative impact on animal welfare and lifesaving capacity. This impact is even greater for organizations operating in endemic areas, where a high percentage of dogs may be infected. Additionally, a prolonged period of exercise restriction during HW treatment is of particular concern for shelter dogs [19], which are already subject to chronic stress [20] and for whom exercise may be the most available and practical form of enrichment. Placing dogs with HWI in foster homes for the duration of their treatment is ideal, but not always feasible. The ability to provide a shorter and less expensive course of heartworm treatment, that is also safe and effective, would be of considerable value to AWOs. The cost and logistics of HW treatment are also important barriers to care for lower-income clients [19]. In conventional private practice and dedicated low-cost veterinary care settings, a modified arsenical treatment protocol could improve owner compliance and decrease owner anxiety engendered by delayed melarsomine treatment.

This retrospective study evaluated modified three-dose melarsomine treatment protocols in a shelter setting and compared them to the AHS protocol.

## Methods

The study examined the clinical records of HW-positive dogs admitted to Toronto Humane Society (THS) from 1 January 2015 to 31 August 2019. Data were retrieved from the PetPoint Data Management System and scanned paper records. Dogs were excluded if they had previously received adulticide treatment, had severe clinical signs of heartworm disease (HWD) prior to M1 (right heart failure or caval syndrome), or had a serious unrelated comorbidity.

### Diagnostics

Heartworm infection was diagnosed as described previously [21]. An initial positive antigen test was confirmed by a second positive antigen test from a different source (e.g., source shelter and THS, or THS and reference laboratory) or by positive identification of microfilariae on direct microscopy or a microfilarial filtration test (MFT). A physical examination by a veterinarian, complete blood count, and biochemistry profile were performed for all dogs with HWI. Thoracic radiographs and other diagnostics were at the attending veterinarian’s discretion.

### Treatment protocol and case management

The AHS guidelines recommend doxycycline 10 mg/kg PO BID from days 1 to 28, and melarsomine on days 60, 90, and 91. As compared with the AHS guidelines, the shelter protocols utilized doxycycline 10 mg/kg SID or BID and varied the time from initiation of doxycycline (day 1) to the first melarsomine injection (M1). Current AHS and THS protocols are shown in Table 1.

**Table 1:** Toronto Humane Society (THS) heartworm treatment protocol compared with 2020 American Heartworm Society (AHS) treatment guidelines. Modifications are shown in italics type. Day 0, day of diagnosis

	AHS guidelines, Nelson et al. [1]	Current THS protocol
Diagnosis	Day 0	Day 0
Macrocyclic lactone	Day 1, then monthly	Day 1, then monthly
Doxycycline	10 mg/kg PO BID, days 1–28	10 mg/kg PO *SID*, days 1–28
Prednisone	Prior to melarsomine if symptomatic At time of M1 (day 61); M2 (day 90) 0.5 mg/kg PO BID first week, 0.5 mg/kg SID second week, 0.5 mg/kg every other day for the third and fourth weeks	Prior to melarsomine *if deemed necessary; dose and duration at clinician discretion* At time of M1 and M2—as per AHS guidelines
Melarsomine	First injection day 61, second and third injections days 90 and 91	First injection *day 14*, second and third injections *days 44 and 45*
Test for microfilariae	Day 120 and 9 months after last melarsomine	*Day 75* and *6 months* after last melarsomine^a^
Antigen test	9 months after last melarsomine	*6 months* after last melarsomine^a^
Protocol complete	12 months from diagnosis	*7.5 months* from diagnosis

^a^Testing 6 months after melarsomine was performed for dogs still in shelter care, or recommended to adopters if adopted earlier

Exercise restriction was implemented from the time of diagnosis, with no off-leash activity allowed. More stringent restrictions were implemented after M1 (shorter and less frequent walks). Activity was then gradually increased 6–8 weeks after M2/3.

Intact dogs that were asymptomatic, or mildly symptomatic with normal thoracic radiographs, were spayed or neutered prior to melarsomine treatment. Dogs were made available for foster or foster-to-adopt placement unless other medical or behavioral conditions required intervention. Detailed counseling was provided to foster guardians, with frequent check-ins. An MFT was performed at least a month after M2/3. If negative, the adoption was finalized for foster-to-adopt dogs. If positive, the MFT was repeated approximately monthly until a negative result was obtained, at which point the adoption was finalized.

### Clinical signs

**Fig. 1:**
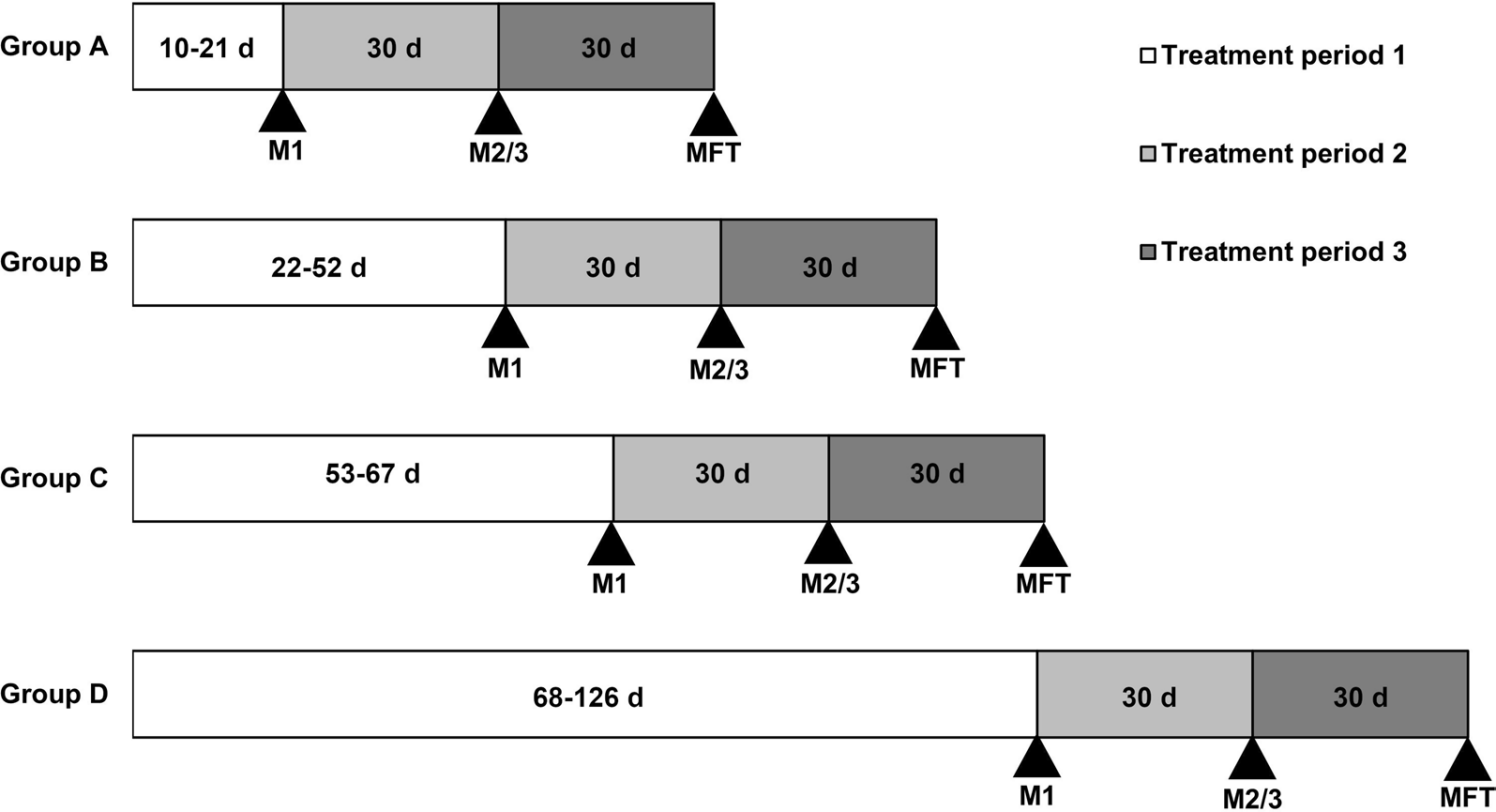
Schematic diagram showing treatment periods and retrospective treatment groups for shelter dogs treated using three-dose melarsomine protocols. Treatment period 1, days from diagnosis to first melarsomine (M1); Treatment period 2, days from M1 to second/third melarsomine (M2/3); Treatment period 3, days from M2/3 to microfilarial filtration test (MFT)

Clinical signs were grouped into three treatment periods (TP): TP1, from diagnosis (day 0) to M1; TP2, from M1 to M2/3; and TP3, from M2/3 to MFT (Fig. 1). Respiratory signs were recorded as present even if they were recorded on only one occasion. They were also recorded as “previously noted” or “new,” in relation to the TP in question. Respiratory signs that required additional intervention were classified as respiratory complications. GI signs during TP1 were recorded.

### Post-adoption survey

Adopters of dogs that had completed treatment for HWI at least 6 months prior were surveyed by telephone.

### Statistical analysis

To compare the current THS protocol to AHS guidelines, the time to M1 was grouped as 14 ± 7 days (Group A) and 60 ± 7 days (Group C) after initiation of doxycycline. Groups that fell outside these intervals were designated as Group B (days 22–52) and Group D (> day 67) (Fig. 1). Data were analyzed using Excel and GraphPad Prism 9.2.0. Quantitative data were compared using the Mann–Whitney *U* test, two-tailed. The Chi-square and Fisher’s exact tests were used to compare categorical variables. Significance was set at *P* < 0.05.

## Results

One hundred and sixty-six HW-positive dogs were identified. Nine were excluded—six had already received adulticide treatment, one had right-sided heart failure, and two had serious unrelated comorbidities—resulting in a sample size of 157. Following M1, hind limb paresis occurred in one dog and hepatopathy in one. These dogs had received M1 on days 16 and 19, respectively. An anaphylactic reaction occurred in one dog after M2; this dog had received M1 on day 36. All three dogs responded to symptomatic treatment. Melarsomine was terminated and topical moxidectin was continued until a negative antigen and MFT. Termination of melarsomine resulted in a sample size of 157 for TP1 and TP2, and 155 for TP3.

Chronic kidney disease (CKD), presumed to be HW-related, was diagnosed shortly after intake in two dogs; both were successfully treated for HWI. One hundred and fifty-three dogs (97%) were fostered for at least part of the HW treatment period, with 118/157 (75.2%) in foster care for > 60% of the treatment duration. Almost all of the dogs were adopted (156/157, 99.4%). One was euthanized for CKD following successful treatment of HWI, after more than a year in foster care.

### Respiratory signs and complications

**Fig. 2:**
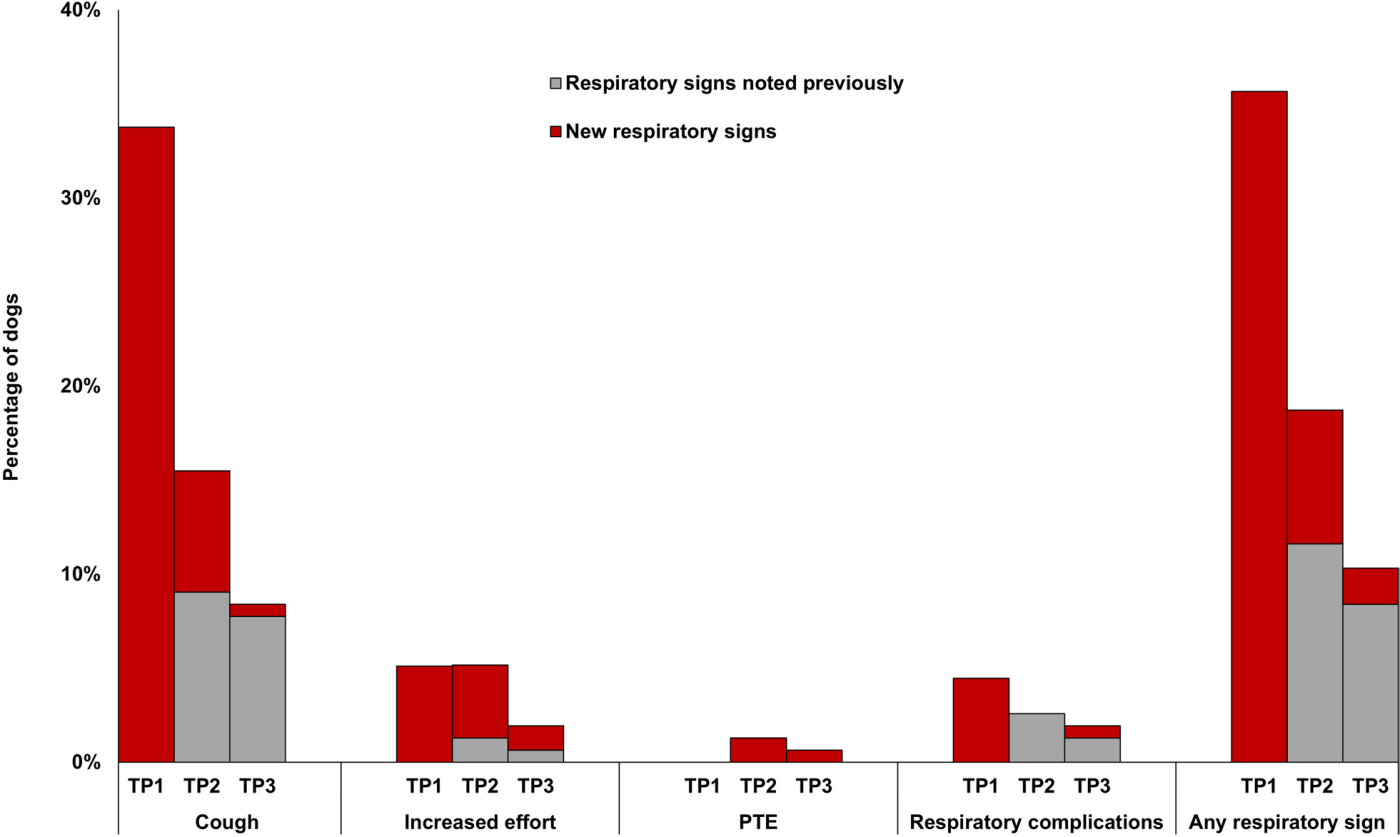
Respiratory signs in 157 shelter dogs treated with three-dose melarsomine. Respiratory complications were defined as respiratory signs requiring additional treatment. *TP* time period, *TP1* initiation of doxycycline to first melarsomine (*n* = 157), *TP2* first melarsomine to second/third melarsomine (*n* = 157), *TP3* second/third melarsomine to follow-up MFT (*n* = 155). *PTE* suspected pulmonary thromboembolism

The most common respiratory sign was coughing, which was most prevalent during TP1 (53/157 dogs; 33.8%) (Fig. 2). Fourteen dogs developed new respiratory signs following melarsomine treatment—11/157 dogs (7.0%) during TP2 and 3/155 (1.9%) during TP3. Respiratory signs were mild in most cases.

**Fig. 3:**
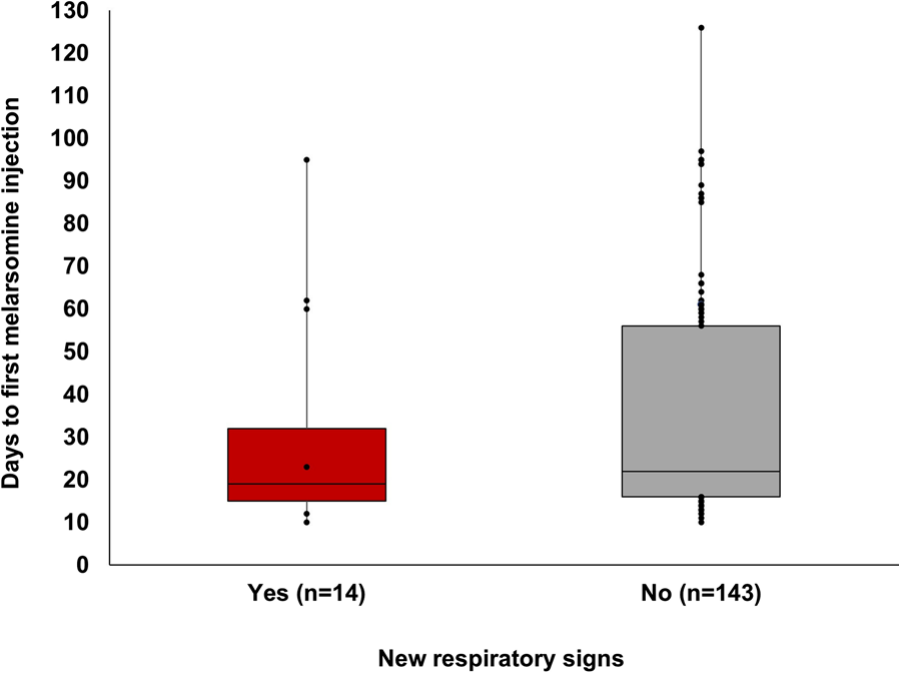
Days to first melarsomine injection after initiation of doxycycline, for dogs with and without new respiratory signs after melarsomine. Data are shown as box plots including median and 25th and 75th percentiles, with whiskers showing range

The time to M1 for dogs with new respiratory signs [median (25th percentile; 75th percentile)] was 19 days (15; 32), and 22 days (16; 56) for dogs without new respiratory signs (Fig. 3). There was no significant difference between these groups (Mann–Whitney *U*-test, *U* = 793, *P* = 0.203).

Seven dogs (4.5%) had respiratory complications during TP1 (Table 2) and were treated with prednisone (5), prednisone and sildenafil (1), and brief hospitalization and antibiotic treatment for suspected bronchopneumonia (1). Five dogs required new treatments for six respiratory complications after melarsomine (Table 2). Respiratory signs had been present during TP1 in four of these dogs. Two dogs had suspected PTE during TP2, and one developed caval syndrome (confirmed by ultrasound) after M1, which resolved without surgical intervention. This dog also developed suspected PTE after M2/3. Three dogs required a short-term increase in prednisone dose or frequency, and two required additional diagnostics and intervention. All five dogs recovered.

**Table 2:** Respiratory  complications, defined as respiratory signs that required additional treatment, in shelter dogs treated for heartworm infection. Italicized text shows time period of onset

Dog #	Group	Days to M1	Doxycycline frequency	Treatment period 1		Treatment period 2		Treatment period 3	
				Problem	Treatment^a^	Problem	Treatment^a^	Problem	Treatment^a^
125	A	19	Once daily	Cough		*PTE*	*Prednisone*	N/A^c^	
29	A	21	Once daily	*Cough*	*Prednisone*	*Caval syndrome*	*Prednisone, CL*	*PTE*	*Prednisone, CL*
82	B	22	Twice daily	*Cough*	*Prednisone*	Cough		Cough	
85	B	23	Once daily	*Cough*	*Prednisone*				
23	B	28	Once daily	Cough, IRE				*Cough, IRE*	*Prednisone*
144	B	30	Once daily	*Cough, H, PHT*	*Prednisone, sildenafil*	Cough	Prednisone, sildenafil		Sildenafil
71	B	34	Twice daily	*Cough*	*Prednisone*				
27	B	46	Once daily	Cough		*PTE*	*Prednisone,* *CL,* O_2_	Cough	CL
39	B	49	Twice daily	*Cough, IRE*	*Prednisone*	IRE			
36	C	60	Twice daily					*IRE*	*Prednisone*
47	D	86	Twice daily	*Cough, IRE*	*Am-C*	Cough		Cough	

*H* hemoptysis, *IRE* increased respiratory effort, *M1* first melarsomine injection, *O*_*2*_ oxygen, *PTE* suspected pulmonary thromboembolism, *PHT* pulmonary hypertension, *CL* clopidogrel

*Am-C* Amoxicillin-clavulanic acid for suspected bronchopneumonia

^a^In addition to standard treatment

^c^Melarsomine discontinued after first melarsomine due to hepatopathy

Seventy-nine dogs received M1 at 14 ± 7 days after initiation of doxycycline (Group A; current THS protocol) and 27 at 60 ± 7 days (Group C; current AHS protocol) (Table 3). Thirty-seven received M1 from days 22 to 52 (Group B) and 14 from days 68 to 126 (Group D). New respiratory signs were recorded for nine dogs in Group A (11%), two in Group B (5.4%), two in Group C (7.4%), and one in Group D (7.1%). There was no statistically significant difference (Fisher’s exact test, *P* = 0.726) between Groups A and C. Due to small sample sizes, statistical comparisons were not made between all four groups. Two dogs in Group A (2.5%), two in Group B (5.4%), and one in Group C (3.7%) developed respiratory complications during melarsomine treatment (Table 2).

**Table 3:** Key results for 157 shelter dogs treated for heartworm infection and retrospectively grouped based on timing to the first melarsomine injection (M1)

Group	n	Days to M1	Doxycycline once daily	Doxycycline twice daily	New respiratory signs	Respiratory complications
A	79	16 (15;19)	79	0	9 (11.4%)	2 (2.5%)
B	37	26 (24; 32)	29	8	2 (5.4%)	2 (5.4%)
C	27	61 (59; 62)	1	26	2 (7.4%)	1 (3.7%)
D	14	94 (86; 95)	0	14	1 (7.1%)	0 (0.0%)
Total	157		109	48		

Days to M1 are shown as median (25th; 75th percentile). Respiratory signs and complications post-melarsomine are shown as number (%)

### Doxycycline—respiratory signs and GI side effects

One hundred nine dogs received 10 mg/kg doxycycline PO SID and 48 received 10 mg/kg PO BID. Twenty-six of 27 dogs in Group C (94%) received BID doxycycline, while all 79 dogs in Group A received SID doxycycline (Table 3). The time to M1 [median (25th percentile; 75th percentile)] for SID doxycycline was 18 days (15; 23), compared with 61 days (57; 86) for BID doxycycline (Mann–Whitney *U*-test, *U* = 107, *P* < 0.0001). New respiratory signs following melarsomine occurred in 10/109 (9.2%) dogs receiving SID doxycycline and 4/48 (8.3%) dogs receiving BID doxycycline, with no significant difference between these groups (Fisher’s exact test, *P*≥0.999).

GI signs prior to M1 were recorded for 40/109 (37%) dogs receiving SID doxycycline and 25/48 (52%) receiving BID doxycycline. This difference was not statistically significant (Fisher’s exact test, *P* = 0.08). There was also no statistically significant difference between SID and BID doxycycline when lethargy and anorexia were included as GI signs.

### Post-adoption survey

Data were obtained for 75 of 153 dogs that qualified for the post-adoption survey (49.0%). Documented antigen test results were available for 44 dogs. Three were tested 2–3 months after M2/3, 14 after 4–8 months, and 27 after ≥ 9 months (mean 16 months, range 2–24 months). Only four dogs were tested 6 or 7 months after melarsomine, as recommended by the shelter. There were two positive results, from dogs tested 2 and 3 months after melarsomine. Both tested negative when retested at 9 and 13 months, respectively.

## Discussion

This study demonstrated the viability of a shorter, less expensive three-dose melarsomine protocol in a shelter setting. The incidence of new respiratory signs following melarsomine treatment did not differ between dogs that received M1 early and those that did not. Respiratory complications during melarsomine treatment were uncommon and typically required little intervention. Compared with AHS guidelines, the current THS protocol shortens the duration of treatment by 6 weeks. In addition to the benefits of earlier parasite death, a shorter treatment period decreases shelter length of stay and the duration of exercise restriction, thereby improving animal welfare and increasing lifesaving capacity. Further, 10 mg/kg doxycycline SID, rather than BID, was not associated with higher incidence of respiratory signs after melarsomine. There was a lower incidence of GI signs during SID doxycycline treatment, although this difference was not statistically significant.

New respiratory signs following melarsomine occurred in 8.9% of all dogs, 11.4% of Group A and 7.4% of Group C. In early clinical trials of melarsomine (without doxycycline), more than 40% of dogs had “pulmonary reactions,” including coughing, gagging, panting, increased respiration and dyspnea [22]. Coughing or gagging alone occurred in 22.2% of dogs with Class 1–3 HWD, and dyspnea in 2.6% [22]. Another study reported mild signs (primarily cough and lethargy) following doxycycline and melarsomine treatment in 4/15 dogs (26.7%) [23]. These dogs all had high parasite burdens on ultrasound examination. A study that included all classes of severity found a high incidence of respiratory complications in dogs receiving doxycycline and three-dose melarsomine (48%), but these were mild in all dogs with Class 1 HWD [24]. Nelson et al. [7] reported respiratory signs post-melarsomine in 6.5% of 47 client-owned dogs that also received doxycycline, and 19% of 47 dogs that did not. Finally, a study that used multiple adjunctive treatments reported coughing and mild dyspnea in only 1/44 (2%) of dogs [25]. Incidence in our study was influenced by the liberal case definition for respiratory signs, and possibly by heavier parasite burdens in a shelter population. The study data did not provide any indication of adult worm burden.

Other than the study that included Class 3 and 4 HWD [24], no deaths attributable to HWD were reported in the recent studies above, as was the case in the current study.

This study was the first to record respiratory signs and complications during different HW treatment periods. A previous clinical study reported, pre-treatment, harsh lung sounds in 17.3%, cough in 16.2%, dyspnea in 1.8%, and PTE in 0.7% [26]. The high pre-melarsomine incidence of signs, particularly cough, in our study might have been caused by clinical manifestations of HWD, but this would not explain why far fewer dogs had respiratory signs in TP2 and TP3, the periods during which respiratory signs might be expected to occur due to rapid adult worm death. Possible reasons are over-reporting before fostering, while dogs were being closely observed by multiple staff members and volunteers; concurrent infectious respiratory disease at admission; reduced respiratory signs after exercise restriction was implemented; and, in rare cases, respiratory reactions to rapid die-off of microfilariae following ML treatment [27, 28].

There is compelling evidence for the benefits of doxycycline in treatment of HWI [1, 5–11]. At least some delay prior to M1 is advisable, in order to allow time for doxycycline to take effect. At issue is the precise amount of time that is optimal. Safe and effective use of melarsomine 30 days after initiation of doxycycline was demonstrated in 76 naturally infected dogs [13]. Most dogs (61%) in that study had clinical HWD, and 17% had a high parasite burden on ultrasound examination. Our results support initiation of melarsomine after only 2 weeks of doxycycline. A full 28-day course of doxycycline was completed in all dogs and remains advisable, based on current knowledge.

The use of doxycycline at 10 mg/kg PO SID, rather than BID, in the current THS protocol was based on a lack of consensus between expert guidelines, absence of studies demonstrating superiority of BID dosing, cost advantages of less frequent dosing, and concerns about GI adverse effects [19]. In one study, doxycycline 10 mg/kg BID was superior to SID dosing, as measured by residual microfilarial *Wolbachia* DNA [14], but negative PCR or antibody tests may not be a necessary end-point for effective clinical reduction of the pro-inflammatory effects of *Wolbachia*. Our study showed no statistically significant differences between SID and BID doxycycline, either for new respiratory signs or for GI signs. However, a higher percentage of dogs treated BID had GI signs. This was consistent with an experimental study in which 4/8 dogs administered 10 mg/kg doxycycline BID had GI side effects compared with 2/8 dogs receiving 5 mg/kg BID [14]. Another study reported GI effects in 6% of dogs treated with doxycycline 10 mg/kg BID [25]. These resolved after reducing the dose to 5 mg/kg BID. Similarly, GI signs necessitated reducing doxycycline to 5 mg/kg BID in 12/50 dogs in an earlier study [24]. As with respiratory signs, the case definitions for GI signs in our study were liberal, with signs being mild and self-limiting in many cases. This most likely contributed to the reported high incidence. Many confounding factors, including stress, diet change, and infectious disease, can cause GI signs in a shelter environment, and microfilarial die-off can also be associated with vomiting or diarrhea [27].

Non-respiratory complications of melarsomine treatment occurred in three dogs in our study. The most commonly reported adverse effects are injection site reactions, coughing/gagging, lethargy, and inappetence, but a wide range of rare complications may occur [24, 29, 30].^1^ Interestingly, injection site reactions were rare in our study (data not shown), possibly because sedation and mu-agonist opioid analgesia are administered prior to injection as part of the shelter treatment protocol.

All antigen tests performed > 3 months after M2/3 were negative in our study. This included 14 dogs tested 4–8 months after M3. This is in agreement with a previous study in which all antigen tests were negative 6 months after a shortened melarsomine protocol [13]. These findings support the absence of a “susceptibility gap” when using MLs, doxycycline, and melarsomine, as previously outlined [12]; however, tests may remain positive beyond 6 months in some successfully treated dogs [1, 24]. Repeat antigen test results were only available for 44 dogs, a surprisingly low proportion and consistent with low retesting rates reported elsewhere [24]. Few adopters tested at the recommended interval after treatment. The missing antigen test data represents a limitation of the study, in that adulticide efficacy was not confirmed in most of the dogs. However, the exceptional efficacy of three-dose melarsomine [1, 31] provides substantial confidence that this treatment was successful.

Other limitations of the study included its retrospective nature, the potential for non-HW-related causes of respiratory and GI signs in a shelter environment, and potential over-reporting of these signs. Clinical signs were reported if they were mentioned even once in the electronic medical record, rather than, as would be more typical in clinic records, if they were enough of a concern to require a consultation or intervention. Case definitions have not been proposed in previous studies that reported respiratory signs during HW treatment [7, 13, 24, 32–34]. These would be helpful for future comparisons between protocols.

## Conclusions

This study provided support for initiating melarsomine after 14 days of doxycycline treatment and for SID doxycycline dosing. Shorter and less expensive treatment protocols can safely increase shelter lifesaving capacity. A shorter period of exercise restriction has substantial quality of life benefits for dogs.

## Data Availability

The datasets are available from the corresponding author on reasonable request.

## Abbreviations

AHS: American Heartworm Society
BID: Twice daily
HW: Heartworm
HWD: Heartworm disease
HWI: Heartworm infection
ML: Macrocyclic lactones
M1, M2, M3: First, second, and third melarsomine injection, respectively
SID: Once daily

## Footnotes

1 https://www.drugs.com/vet/immiticide.html, https://www.drugs.com/vet/diroban.html—accessed 5 October 2022.
